# Advances on adaptive immune responses affected by infectious bursal disease virus in chicken

**DOI:** 10.3389/fimmu.2023.1330576

**Published:** 2024-01-10

**Authors:** Tao Zhang, Suyan Wang, Yongzhen Liu, Xiaole Qi, Yulong Gao

**Affiliations:** ^1^ Avian Immunosuppressive Diseases Division, State Key Laboratory for Animal Disease Control and Prevention, Harbin Veterinary Research Institute, the Chinese Academy of Agricultural Sciences, Harbin, China; ^2^ World Organization for Animal Health (WOAH) Reference Laboratory for Infectious Bursal Disease, Harbin Veterinary Research Institute, the Chinese Academy of Agricultural Sciences, Harbin, China; ^3^ Jiangsu Co-Innovation Center for the Prevention and Control of Important Animal Infectious Disease and Zoonosis, Yangzhou University, Yangzhou, China; ^4^ National Poultry Laboratory Animal Resource Center, Harbin, China

**Keywords:** IBD, IBDV, immunosuppression, adaptive immune response, lymphocytes

## Abstract

Infectious bursal disease (IBD) is an acute, highly infectious, and immunosuppressive disease caused by the infectious bursal disease virus (IBDV), which interferes with the immune system, causes hypoimmunity and seriously threatens the healthy development of the poultry industry. Adaptive immune response, an important defense line of host resistance to pathogen infection, is the host-specific immune response mainly mediated by T and B lymphocytes. As an important immunosuppressive pathogen in poultry, IBDV infection is closely related to the injury of the adaptive immune system. In this review, we focus on recent advances in adaptive immune response influenced by IBDV infection, especially the damage on immune organs, as well as the effect on humoral immune response and cellular immune response, hoping to provide a theoretical basis for further exploration of the molecular mechanism of immunosuppression induced by IBDV infection and the establishment of novel prevention and control measures for IBD.

## 1 Introduction

Infectious bursal disease (IBD) is an acute, highly contagious, and immunosuppressive disease caused by the infectious bursal disease virus (IBDV) (1). IBDV has been popular for over 70 years, bringing huge economic losses to the global poultry industry. Belonging to the genus Avibirnavirus within the Birnaviridae family, IBDV is categorized as two distinct serotypes: serotypes I and II. Notably, only serotype I possesses pathogenicity towards chickens (2). According to the antigenicity and pathogenicity, serotype I IBDV can further be divided into classical IBDV (cIBDV) (3), variant IBDV (varIBDV) (4), and very virulent IBDV (vvIBDV) (5, 6). IBDV mainly infects immature IgM^+^ B lymphocytes in the bursa and induces the atrophy of the targeted organ bursa and the damage of B lymphocytes (7), which reduces the ability of immune response (8), leading to serious mixed infections and secondary infections, results in increased morbidity and mortality in chickens (9), and reduces the immunity effects induced by other avian vaccines (10–12).

The integrity of both innate and adaptive immune is essential for defending against the pathogenic invasion (13). IBDV infection can destroy the immune system of chickens which further results in the inability to resist the pathogen. The innate immune response acts as the host’s first line of defense against broad-spectrum pathogen infection and exerts its antiviral effects through natural immune cells and immune molecules (14), and how IBDV infection suppresses the innate immune response has been extensively resolved. For example, dsRNAs can be recognized by the pattern recognition receptor MDA5 and trigger downstream signal transduction pathways to induce type I interferon (IFN-I) production (15). In contrast, the viral protein VP3 of IBDV can competitively bind dsRNA with MDA5, affecting the host’s innate immune response and promoting IBDV replication during the infection process (16). As an important line to resist pathogen infections, the adaptive immune response includes humoral immunity and cellular immunity and is mainly mediated by T and B lymphocytes in host-specific immune responses against specific pathogens. IBDV infection mainly attacks the immune organs and immune cells, resulting in the atrophy of the bursa, necrosis of B lymphocytes, and impaired activation of T lymphocytes (17, 18), severely affecting the body’s adaptive immune response.

This review mainly focuses on the impact of IBDV infection on host adaptive immune response, from the perspective of the effects of IBDV infection on the central immune organs and peripheral immune organs, as well as the effects on humoral and cellular immune responses. Therefore, these summaries may provide theoretical references for exploring the causes of immunosuppression and the prevention and control measures of IBD.

## 2 Effects of IBDV infection on the immune organs

IBDV infection can not only seriously damage the central immune organs (bursa, thymus and bone marrow), but also affect peripheral immune organs such as the spleen, gut-associated lymphatic tissue (GALT), Gland of Harder (GH), severely damaging the function of T and B lymphocytes and further affecting the adaptive immune response (19–22). These finding is closely related to immunosuppression in chickens caused by IBDV infection. Therefore, a comprehensive analysis of the effects of IBDV infection on the immune system will be more important for the scientific prevention and control of IBD infection.

### 2.1 Effects of IBDV infection on the central immune organs

#### 2.1.1 Effect of IBDV infection on the bursa

The damage to the bursa is most obvious after an IBDV infection. Yellow exudate on the serous membrane of the bursa is found in the early stage of vvIBDV infection. As the course of viral infection progresses, the bursa appears necrotic foci, spot-like or hemorrhagic bleeding on the mucosal surface, presenting a classic “purple grape” appearance (8, 19–21, 23). Histopathological results show that the follicles are atrophied after vvIBDV infection, with a large reduction of lymphocytes due to necrosis, local medullary vacuolation, significant neutrophil infiltration and interstitial hyperplasia, accompanied by erythrocyte infiltration, resulting in bursa of atrophy and bleeding (19).

**Figure 1:**
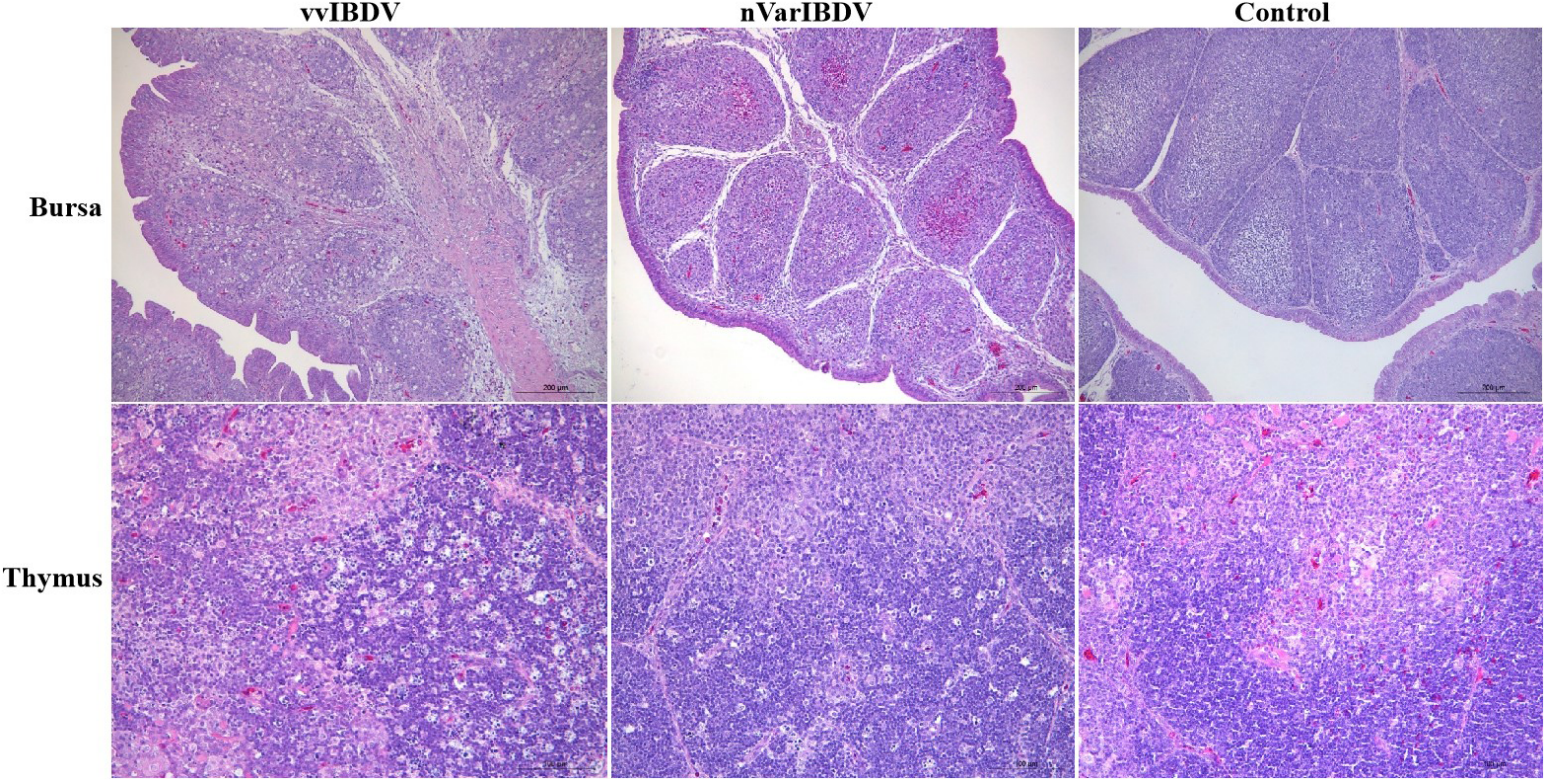
Pathological change with vvIBDV HLJ0504 and nVarIBDV SHG19 infection of bursa of Fabricius and thymus. After vvIBDV HLJ0504 and nVarIBDV SHG19 infection of bursa of fabricius for 4 days, follicular atrophy, necrosis of lymphocytes, infiltration of xenophil cells and interstitial hyperplasia were observed. The pathological results of thymus showed that lymphocyte necrosis decreased and macrophages proliferated after HLJ0504 and SHG19 infection. No significant abnormality was found in the blank control group.

In contrast to vvIBDV, varIBDV infection was originally described in the United States and typically causes severe bursa lesions (24). Recently, a novel type of varIBDV (nVarIBDV), belonging to the A2dB1 genotype, was wildly prevalent in China, which is different from the early North American varIBDV (A2aB1, A2bB1 and A2cB1 genotype) (21, 22). Besides, we found the number of medullary lymphocytes in the follicle of bursa was significantly reduced with heterophil infiltration and interstitial hyperplasia in SPF chickens after nVarIBDV infection (23) (**Figure 1**), which is similar to the histopathological damage caused by vvIBDV infection after 4 days post infection.

At 4 days post-infection of vvIBDV and nVarIBDV, the bursa showed follicular atrophy, necrosis of lymphocytes, infiltration of xenophil cells and interstitial hyperplasia, and the pathological results of thymus showed that lymphocyte necrosis decreased and macrophages proliferated. No significant abnormality was found in the Mock group.

#### 2.1.2 Effect of IBDV infection on the thymus and bone marrow

Similar to the damages in the bursa, the thymus is swollen with hemorrhagic spots in the early stage of vvIBDV infection, and severe atrophy is seen with the course of viral infection progresses. When infected with vvIBDV, the chickens show acute, necrotizing thymic pathological changes in the thymus, manifested by a small number of cavities in the cortex, congestion and infiltration of eosinophilic granulocytes, the decrease of cortical thymocytes, and massive necrosis of lymphocytes (**Figure 1**). The thymus also displays lymphocyte degeneration and necrosis, with mild reduction of thymic cortical lymphocytes and mild proliferation of macrophages in nVarIBDV group (23) (**Figure 1**). The thymus serves as the primary site of T lymphocyte maturation, and thus IBDV infection may interfere with the T lymphocyte-mediated adaptive immune response (25, 26). Up to now, no direct evidence shows pathological changes after IBDV infection in bone marrow. Yang et al. demonstrate that IBDV infection impairs the maturation and function of chicken bone marrow-derived dendritic cells (chBM-DCs), suggesting that IBDV infection may take chBM-DCs as the additional target cells, and is related tightly with bone marrow (27). IBDV infection induces damage to the central immune organ and further triggers an invalid specific immune response.

### 2.2 Effects of IBDV infection on peripheral immune organs

#### 2.2.1 Effect of IBDV infection on the spleen

IBDV also has a detrimental effect on peripheral immune organs. When infected with vvIBDV and varIBDV, chickens show enlarged, congested spleens with a dark brown color (23, 28). When the chicken infected with vvIBDV, the spleen shows necrosis and disintegration of lymphocytes and a significant reduction in the number of lymphocytes, resembling the pathological histological changes of the bursa. The spleen contains a large number of mature B lymphocytes, responsible for inducing the initial immune response in response to foreign antigens. Therefore, IBDV infection is speculated to damage the spleen, resulting in impaired function of mature B lymphocytes (8, 29), which is likely closely related to antibody production and affects the host’s adaptive immune response to pathogens. Some studies have demonstrated that IBDV infection can significantly reduce serum antibody titers induced by avian commercial vaccines. For example, Our studies find that nVarIBDV infection can suppress the titer of avian influenza virus (AIV) antibodies against both H5 and H7 vaccinated with a recombinant AIV bivalent inactivated vaccine (H5+H7) (21, 30) and decrease the HI titer of Newcastle disease vaccine (LaSota strain) antibodies by about 23% in commercial broilers and laying hens (11). However, whether spleen damage is the main cause of immune failure needs further investigations in IBDV infection process.

#### 2.2.2 Effect of IBDV infection on the gut-associated lymphoid tissues

Gut-associated lymphoid tissue (GALT) consists of lymphoid nodules, free lymphoid tissue, plasma cells, and mucosal intraepithelial lymphocytes. Cecal tonsils (CT) are the largest intestinal-associated lymphoid tissue in the avian GALT (31). vvIBDV infection results in slight swelling, congestion, and internal hemorrhage of the cecal tonsils. While histopathological results showed that the glandular ducts of the cecal tonsils in chickens are structurally intact and the lymphoid nodules are structurally clear in the pre-infection stage of vvIBDV infection. There is massive necrosis and lysis of lymphocytes within the lymphoid nodules and the diffuse zone of the cecal tonsils, and with reticulocyte hyperplasia, heterophilic granulocyte infiltration, as well as a decrease in the number of B lymphocytes with the course of viral infection progresses (32). Although we have found a high replication titer of varIBDV in the cecal tonsils, there are no reports of pathological changes in the cecal tonsils caused by varIBDV (23). IgA is the most common immunoglobulin in mucosal tissues and is an important immune defense against invasion of intestinal pathogens, and vvIBDV infection causes a decrease in the number of IgA^+^ cells in the cecum tonsils (33). Thus, IBDV infection can cause damage to gut-associated lymphoid tissues and lead to dysregulation of the gut microbiota, which may also contribute to a higher susceptibility of infected birds to pathogens invading the gut (32).

#### 2.2.3 Effect of IBDV infection on the Gland of Harder

Gland of Harder (GH), a peripheral organ of immunity, is more developed in avian species. After chickens infected with vvIBDV, GH shows slightly swollen with surface congestion, and histopathological results of GH reveal intracytoplasmic vesicle formation in the epithelial cells of the adenoducts, some epithelial cells detachment, and some lymphocytes with nuclei condensation, fragmentation, necrosis, accompanied by plasma cell necrosis and heterophilic granulocyte infiltration (34). Therefore, the function of GH is weaken caused by IBDV in infected chickens. In conclusion, IBDV infection can not only severely damage the central immune organs, but also affect the peripheral immune organs or functions, which ultimately leads to the dysfunction of the immune system and seriously affects the adaptive immune response.

## 3 Effect of IBDV infection on the humoral immune response

The humoral immune response is an immunity protection mechanism in that plasma cells produce antibodies to resist pathogens. B lymphocyte surface receptors (BCR) bind to antigenic peptides and further activate B lymphocytes to proliferate and differentiate into memory B lymphocytes and effector B lymphocytes (plasma cells), which then secrete antibodies (35, 36). IBDV mainly infects the immature IgM^+^ B lymphocytes in the bursa and causes severe death of B lymphocytes, which might subsequently hinder the development and effector function, consequently restrain the humoral immune response (7, 37).

### 3.1 Effect of IBDV infection on the programmed cell death of B lymphocyte

**Figure 2:**
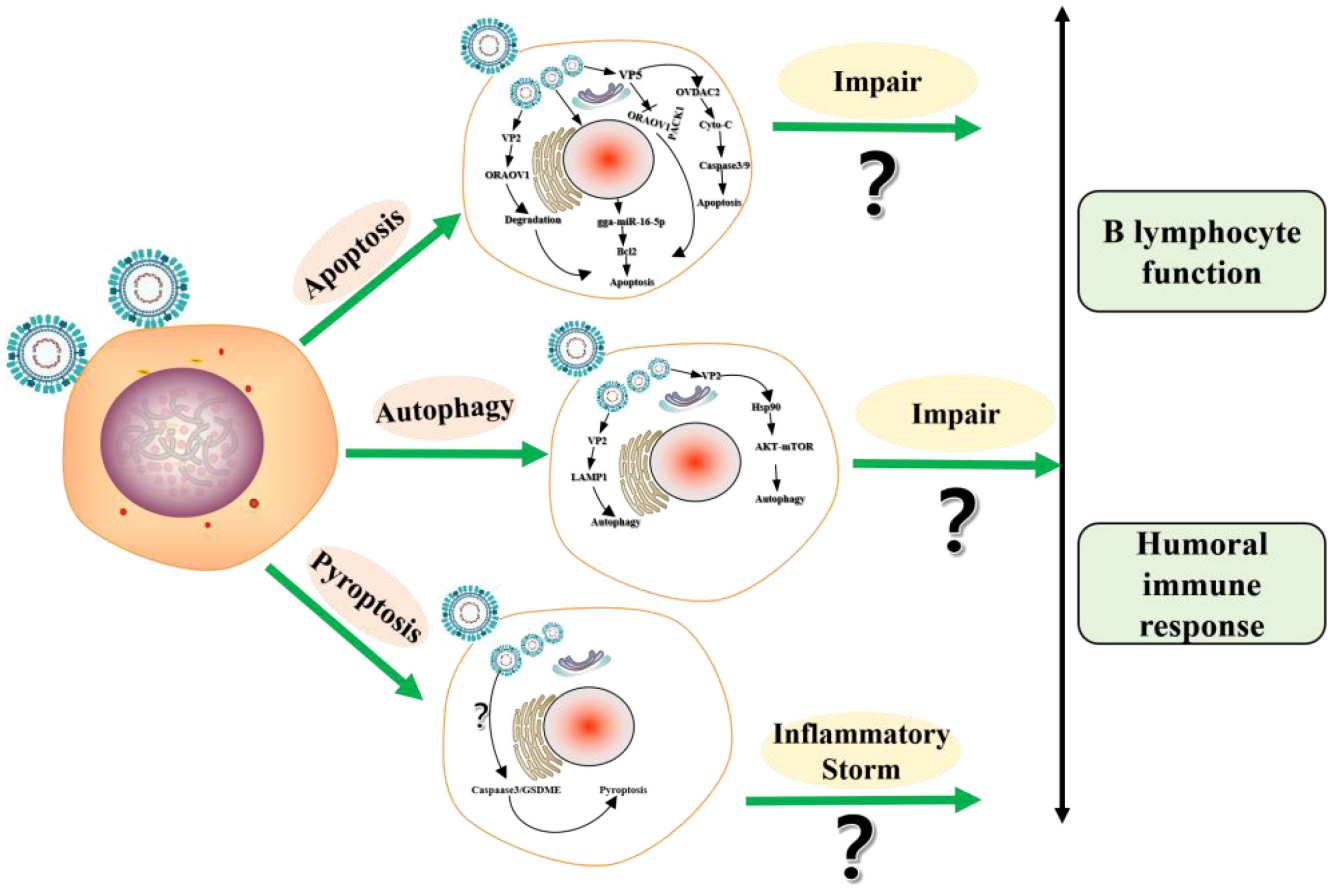
The schematic diagram of programmed cell death (PCD) induced by IBDV.

Both vvIBDV and varIBDV infection can affect the integrity of B lymphocytes in the bursa, further resulting in programmed cell death (PCD), reduced number, and impaired function, which in turn leads to severe atrophy of the bursa (7). As the ways of PCD, the studies on apoptosis and autophagy in the process of IBDV infection have been reported, while studies on pyroptosis does not (38–42) (**Figure 2**). IBDV infection induces apoptosis through various pathways. The viral protein VP2 is the first protein identified as an apoptosis-inducing protein in IBDV infection, and it induced apoptosis by directly interacting with the anti-apoptotic molecule Oral Cancer overexpression protein 1 (ORAOV1) for degradation (39). Subsequently, VP5 may be another protein inducing apoptosis during IBDV infection. Namely, VP5 can interact with Voltage-dependent Anion Channel 2 to promote the release of cytochrome c and the activation of caspase-3 and caspase-9 (40) and can activate apoptosis by preventing the interaction of the anti-apoptotic protein Receptor for Activated C Kinase1 and ORAOV1 (41). In addition, IBDV infection induces up-regulation of non-coding RNAs (microRNAs, miRNAs) gga-miR-16-5p, which can target and inhibit the expression of the anti-apoptotic protein Bcl-2, and enhances the apoptosis induced by IBDV (42).

Cellular autophagy, one of the modes of PCD, is prevalent in the viral infection process. The phenomenon of cellular autophagy has been widely demonstrated during IBDV infection, and the viral protein VP2 plays an important role in IBDV-induced autophagy. Wang et al. find that IBDV can induce autophagic lysosome formation, and autophagic lysosome-associated membrane glycoprotein 1 colocalizes with viral proteins to promote cell lysis and IBDV maturation and release (43). Hu et al. also find that IBDV induces cellular autophagy, and subsequently viral protein VP2 binds to heat shock protein 90 dependent on the AKT-mTOR pathway to induce cellular autophagy (44).

Pyroptosis is also a way of PCD. During the process of pathogen infection, pyroptosis mainly mediates the activation of various Caspase families including Caspase-1, Caspase3 etc, which causes the shear and polymerization of gasdermin family members to result in cell perforation, cell death, and inflammatory response (45–47). When chickens infected with vvIBDV and varIBDV, the bursa induces inflammatory cell infiltration and massive release of inflammatory cytokines, resulting in an “inflammatory storm” in chickens (46). Therefore, the formation of the strong inflammatory response induced by IBDV may be closely related to cellular pyroptosis, but further studies are still needed (4). In conclusion, IBDV infection induces the PCD of B lymphocytes in various ways and may further impair B lymphocyte function.

### 3.2 Effect of IBDV infection on the development of B lymphocytes

The bursa is critical to normal B lymphocyte development in birds. During embryonic development, B cell precursors migrate from the bone marrow to the bursa, where they are induced to increase antigen-dependently (48). After maturation, B lymphocytes migrate from the bursa to the peripheral lymphatic organs. Previous studies have shown that vvIBDV and varIBDV infect bursa and seriously destroy IgM ^+^ B lymphocytes, thus affecting their development (2, 8, 23). There are five main cell clusters (B lymphocyte, T lymphocyte, DCs, epithelial cells, and fibroblast cells) in the bursa that has been proved in the previous research by Yang (36). Among these clusters, the B lymphocyte, especially the IgM^+^ B lymphocyte, is severely damaged upon IBDV infection (36). In addition, some reports find that chicken sIgMλ light chains specifically interact with IBDV *in vitro*, and the binding of IBDV to DT40 cells can be inhibited by sIgM-specific monoclonal antibodies (49), suggesting that sIgM as a binding site participates in IBDV infection. Therefore, the above results may reveal why IBDV mainly invades IgM^+^ B lymphocytes. IBDV destroys IgM^+^ B lymphocytes in the bursa to reduce their number and impair their function, thus affecting B lymphocyte development and maturation.

### 3.3 Effect of IBDV infection on the antigen recognition of B lymphocytes

B lymphocytes are the primary effector cells of the humoral immune response. The ability to capture, process, and deliver antigens to T lymphocytes is essential for the humoral immune response (50). In the process of the host adaptive immune response induced by viral infection, B lymphocytes recognize antigens through BCR, which subsequently requires the recruitment of cytoplasmic protein tyrosine kinase (PTK) to trigger the BCR signal pathway, thereby inducing B lymphocyte activation and completing antigen recognition (51, 52). The JAK-STAT and PTK Lyn-STAT pathways are two independent cascades of the BCR signal pathway (51, 52). Transcriptome profiling of DT40 cells infected with vvIBDV shows that the expression of Janus Kinase 1 and Protein Tyrosine Kinase 2 decreases at 12, 18, and 24 hours post-infection (hpi), which further attenuates the triggering of the BCR signal pathway and thus affects the BCR antigen recognition signal pathway (53). The above results indicate that IBDV infection may affect the BCR signal pathway. However, the current studies only show the transcription level changes of the related gene of the BCR signal pathway, and more studies are needed to determine the expression levels of antigen recognition-associated proteins and how BCR performs antigen recognition and conducts signals.

### 3.4 Effect of IBDV infection on the antigen presentation of B lymphocytes

B lymphocytes act as antigen-presenting cells to recognize antigen (Ag) specifically via the BCR. Subsequently, the BCR-Ag complex is internalized and transported to the region rich with specialized MHC Class II molecules for presenting to specific CD4^+^T lymphocytes (54). These CD4^+^ T-B interactions provide the necessary activation signals for B lymphocyte affinity maturation and differentiation into memory B lymphocytes or plasma cells (53, 55). It has been shown that vvIBDV infection affects the assembly of the nascent peptide chain and translocation of MHC class II molecules. In addition, vvIBDV infection can also down-regulate the expression levels of MHC class II molecules, such as CD8a, CD74, and BCL6, as well as the expression level of Calmegin, which is responsible for assembling MHC class II molecules (52). Thus, vvIBDV infection can affect the antigen presentation process by influencing the expression and assembly of MHC class II molecules in B lymphocytes. However, the visualization of antigen recognition and presentation processing, the assembly process of MHC class II molecules, and the specific interaction between B lymphocytes and CD4^+^ T cells, still need to be further studied.

## 4 Effect of IBDV infection on the cellular immune response

The cellular immune response is mainly mediated by T lymphocytes, whose functions have recently been compromised in IBDV-infected chickens (56). Although the reason for impairing T lymphocyte function is not well known, the direct lytic effect of IBDV on thymus cells or preponderance of immature, immunologically unreactive lymphocytes has been suspected to be responsible (26). The current studies on the effect of cellular immune response mainly focus on the effect on T lymphocyte tissue distribution and migration induced by IBDV infection. Cellular immune responses play an important role on pathogen clearance, but describing the specific mechanism of cellular immune response activation after IBDV infection is difficult, which requires further studies.

### 4.1 Effect of IBDV infection on the development of T lymphocytes

The thymus is the main site of T lymphocytes maturation through negative-positive selection (57). In the early stage of vvIBDV and varIBDV infection, the thymus underwent marks atrophy and widespread apoptosis of thymocytes, and the loss of thymic cortical cells suggested the destruction of T lymphocytes (58, 59). DCs are important for the activation of immature and the proliferation of T lymphocytes, and viral infection induces high expression levels of CD40 and CD86 molecules on the DCs surface and high proliferation capacity of T lymphocytes (26). Furthermore, inactivated IBDV is more effective in stimulating primary T cell responses compared to IBDV infection, possibly due to the higher expression of the co-stimulatory molecules CD40 and CD86 on inactivated-IBDV stimulated-DCs (26). Therefore, IBDV infection may inhibit the proliferative capacity and subsequent development of T lymphocytes.

### 4.2 Effect of IBDV infection on the migration of T lymphocytes into the bursa

Studies have demonstrated that vvIBDV infection is accompanied by the migration of T lymphocytes into the bursa (59). After IBDV infection, T lymphocytes are first detected in the bursa at 4 days post-infection (dpi), and their number increases up to 65% at 7 dpi (60). Some reports show that intrabursal T lymphocytes promote bursal tissue damage and delay tissue recovery by releasing cytokines and cytotoxic effects (60). Therefore, the excessive migration of T lymphocytes may lead to strong inflammatory responses and cause pathologic changes during the IBDV infection process, and the migration of T lymphocytes into the bursa can be considered a marker of the course of IBDV infection (61). Other studies have shown that CD4^+^ T lymphocytes may migrate from the cecal tonsils to the bursa, resulting in a decrease in the number of CD4^+^ T lymphocytes in the cecal tonsils and an increase in the number in the bursa after vvIBDV infection (59). Additionally, the number of CD4^+^ CD25^+^ T lymphocytes decreases in the thymus, while increasing in the bursa, suggesting that CD4^+^ CD25^+^ T lymphocytes infiltrate the bursa along with CD4^+^ T lymphocytes after IBDV infection (62). Consequently, IBDV infection triggers the migration of T lymphocytes from their origin tissues into the bursa, causing a strong inflammatory response and histopathological damage in the bursa.

### 4.3 Effect of IBDV infection on the activation of T lymphocytes

IBDV infection induces significant changes in T lymphocyte subsets. CD4^+^ T lymphocytes (Th cells) are divided into Th1, Th2, Th17, Treg (T regulatory) and Tfh (follicular T helper) cells, according to the cytokines they secrete, and play functions on activation of lymphocytes (63, 64). For example, Th1 cells primarily secrete cytokines such as IL-2, IFN-γ, and TFN-β, which mediate cellular immune response (65). Th2 cells secrete IL-4, IL-5, IL-6, IL-10, and IL-13, which mainly regulate the humoral immune response (66, 67). The characteristic CD4^+^T cell subsets after IBDV infection have not been adequately identified. Previous studies have shown that CD4^+^ T lymphocytes migrate into the bursa and have found that IBDV infection can increase the proportion of CD4^+^ T lymphocytes in the bursa and spleen, as well as the expression levels of IFN-γ, IL-10, and the T lymphocyte immune checkpoint receptor LAG-3 in the bursa (59). VvIBDV infection induces the activation of T lymphocytes in the bursa, while over-activated T lymphocytes enhance the expression level of IFN-γ, which subsequently impairs the function of lymphocytes and leads to T lymphocytes immunosuppression (68, 69). The higher ratio of pro-inflammatory to anti-inflammatory factors can promote pathological changes in tissues and seriously interfere with the immune response mediated by inflammatory cytokines during IBDV infection in chickens (70–72) (**Table 1**). Thus, cytokines secreted by CD4^+^ T lymphocytes may exacerbate the damage to the bursa. Besides, CD4^+^ T lymphocytes may also express the cytolyzing molecule perforin to directly kill lymphocytes (59, 60, 73, 74). Therefore, the high expression level of cytokines induced by IBDV infection may influence the activation and function of T lymphocytes. Although there are no reports of nVarIBDV affecting T cell activation, we speculate that varIBDV may induce T lymphocytes dysfunction, according to our studies on nVarIBDV-induced immunosuppression (30).

**Table 1:** Changes of cytokine transcription levels after IBDV infection.

Cytokine	Types	Strain type	Changes after IBDV infection	Organization/Cell	Reference
IL-Iβ	pro-inflammatory	cIBDV	Up-regulation	Bursa of fabricius	(70)
IL-6	pro/anti-inflammatory	vvIBDV	Up-regulation	DT40 cells	(23, 71)
IL-8	pro-inflammatory	vvIBDV	Up-regulation	DT40 cells	(23, 71)
IL-10	anti-inflammatory/ immunomodulation	vvIBDV	Up-regulation	Bursa of fabricius	(59)
IL-17	pro-inflammatory	vvIBDV	Up-regulation	Bursa of fabricius	(71)
IL-18	pro-inflammatory	vvIBDV/nVarIBDV	Up-regulation	DT40 cells	(23)
IL-2	Th1	vvIBDV	Up-regulation	Bursa of fabricius	(72)
IL-4	Th2	vvIBDV	Up-regulation	Bursa of fabricius	(72)
IL-5	Th2	vvIBDV	Up-regulation	Bursa of fabricius	(72)
IL-12	Th1	vvIBDV	Up-regulation	Bursa of fabricius	(72)
LAG-3	T cell checkpoint receptor	attenuated IBDV strain	Up-regulation	Bursa of fabricius	(59)
IFN-γ	immunomodulation	vvIBDV	Up-regulation	Bursa of fabricius/Spleen	(59, 72)
CTLA-4	Immune checkpoint	attenuated IBDV strain	Up-regulation	Cecal tonsils	(59)

## 5 Conclusions and perspectives

IBDV, especially vvIBDV and varIBDV, can cause severe immunosuppression in chickens, interfere with the immune system and result in economic losses. Besides, vvIBDV and nVarIBDV are the two most prevalent strains of IBDV in China and it is urgent to explore more effective measures for IBD. As the main immunosuppressive pathogen, IBDV attacks the immune organs and immune cells. Efficient prevention and control measures mainly rely on elucidating the adaptive immune response regulatory mechanism of IBDV infection. Therefore, from the immunological perspective, based on current studies of two prevalent IBDV strains, we summarize the effects of IBDV infection on immune organs (central and peripheral organs) and immune responses (humoral and cellular immune responses) in detail.

However, due to the lack of experimental tools, techniques, and basic immunological knowledge in birds, how IBDV affects the adaptive immune response and the specific molecular mechanisms still need to be clarified. Adaptive immunity is stringently regulated by T and B lymphocytes, which facilitate pathogen-specific immunologic effector pathways, the generation of immunologic memory, and the regulation of host immune homeostasis. For instance, the detailed molecular mechanisms by which IBDV infection hinders B lymphocyte development, affects the presentation of B lymphocyte antigens, induces T lymphocytes to infiltrate into the bursa, and activates T lymphocytes are still unclear. Elucidating of molecular mechanisms and solving these problems will help determine the overall impact of IBDV on the immune response, clarify the molecular mechanisms of immunosuppression induced by IBDV, and develop more efficient preventive and control measures.

## Author contributions

TZ: Writing – original draft, Writing – review & editing, Conceptualization. SW: Conceptualization, Funding acquisition, Project administration, Writing – original draft, Writing – review & editing. YL: Conceptualization, Writing – review & editing. XQ: Writing – review & editing. YG: Conceptualization, Writing – original draft, Writing – review & editing, Funding acquisition, Project administration.
